# Genetic associations of the response to inhaled corticosteroids in asthma: a systematic review

**DOI:** 10.1186/s13601-018-0239-2

**Published:** 2019-01-09

**Authors:** Ozlem Keskin, Niloufar Farzan, Esra Birben, Hayriye Akel, Cagatay Karaaslan, Anke H. Maitland-van der Zee, Michael E. Wechsler, Susanne J. Vijverberg, Omer Kalayci

**Affiliations:** 1 Paediatric Allergy and Immunology Department, School of Medicine, Gaziantep University, Gaziantep, Turkey; 2 Department of Respiratory Medicine, University of Amsterdam, Amsterdam UMC, Meibergdreef 9, Amsterdam, Netherlands; 3 Pediatric Allergy and Asthma Unit, Hacettepe University School of Medicine, 06100 Ankara, Turkey; 4 Department of Molecular Biology, Faculty of Sciences, Hacettepe University, Ankara, Turkey; 5 Department of Pediatric Respiratory Medicine and Allergy, University of Amsterdam, Amsterdam UMC, Meibergdreef 9, Amsterdam, Netherlands; 6 Department of Medicine, National Jewish Health, Denver, CO USA

**Keywords:** Inhaled corticosteroids, Asthma, Genetics, Genomics, GWAS

## Abstract

There is wide variability in the response to inhaled corticosteroids (ICS) in asthma. While some of this heterogeneity of response is due to adherence and environmental causes, genetic variation also influences response to treatment and genetic markers may help guide treatment. Over the past years, researchers have investigated the relationship between a large number of genetic variations and response to ICS by performing pharmacogenomic studies. In this systematic review we will provide a summary of recent pharmacogenomic studies on ICS and discuss the latest insight into the potential functional role of identified genetic variants. To date, seven genome wide association studies (GWAS) examining ICS response have been published. There is little overlap between identified variants and methodologies vary largely. However, in vitro and/or in silico analyses provide additional evidence that genes discovered in these GWAS (e.g. *GLCCI1*, *FBXL7*, *T gene*, *ALLC*, *CMTR1*) might play a direct or indirect role in asthma/treatment response pathways. Furthermore, more than 30 candidate-gene studies have been performed, mainly attempting to replicate variants discovered in GWAS or candidate genes likely involved in the corticosteroid drug pathway. Single nucleotide polymorphisms located in *GLCCI1*, *NR3C1* and the *17q21* locus were positively replicated in independent populations. Although none of the genetic markers has currently reached clinical practise, these studies might provide novel insights in the complex pathways underlying corticosteroids response in asthmatic patients.

## Introduction

Affecting up to 18% of the world’s population, asthma is a chronic airway disease and inhaled corticosteroids (ICS) are the preferred first-line treatment for persistent asthma [1]. However, there is a wide variability in response to ICS [2–4] such that up to 35–40% of the patients receiving ICS for 8–12 weeks do not show significant improvement in lung function [2, 3]. Furthermore, 10% of the asthmatic patients on maintenance ICS treatment may remain symptomatic or at high risk of asthma attacks in spite of the regular use of this medication [5]. In addition to poor adherence to medication, continuing noxious environmental exposures, misdiagnosis, and truly steroid refractory disease, genetic variation might also be an important factor influencing treatment response in patients with asthma [4, 6–8]. Approximately 70% of the variance in ICS response is suggested to be due to genetic factors [4, 9].

Over the past three decades, researchers have investigated the relationship between a large number of genetic variations and response to ICS by performing pharmacogenomic studies. The main aim of these studies was to identify genetic markers that could help physicians optimize asthma treatment. The high number of publications has enabled systematic literature reviews that summarize the findings of the studies and evaluate the potential clinical relevance of the findings. One of these systematic literature reviews was published by Farzan et al. [10]. This study reviewed pharmacogenomics studies of ICS and leukotriene modifiers that were published between 1999 and 2015. Furthermore, SNPs that were positively replicated at least once in an independent population were discussed in detail. Despite the large number of pharmacogenomics studies, there is still no genetic marker used in clinical practice to optimize asthma treatment with ICS. However, due to the rapid decrease in the costs of genotyping and emergence of advanced genotyping technologies, efforts are still ongoing to replicate previously identified markers and/or identify new genetic markers by performing Genome-wide association studies (GWAS). One of the important issues of single nucleotide polymorphisms (SNPs) that are identified by GWAS is that their function and relation to the disease/trait of interest is often not clear and therefore might inhibit further progression towards clinical implementation. This has encouraged researchers to perform in vitro and/or in silico studies to unravel the potential role of the SNPs/genes in the asthma-treatment response pathway. Since the field is rapidly evolving, this follow-up systematic review aims to (1) provide a summary of the pharmacogenomics studies on ICS published after 2015 and (2) provide the latest insight into the potential functional role of the identified genes/variants identified in GWAS, in order to provide an overview of the latest promising clinical markers.

## Methods

Articles published from 1995 January through the end of August 2018 were searched in PubMed using three keywords. Conference abstracts, articles assessing adverse drug effects and articles that had not been published in English were excluded. First, we eliminated articles from the titles, and then by abstracts. Then the remaining articles were read in full. We also screened review articles for possible missed publications.

**Fig. 1:**
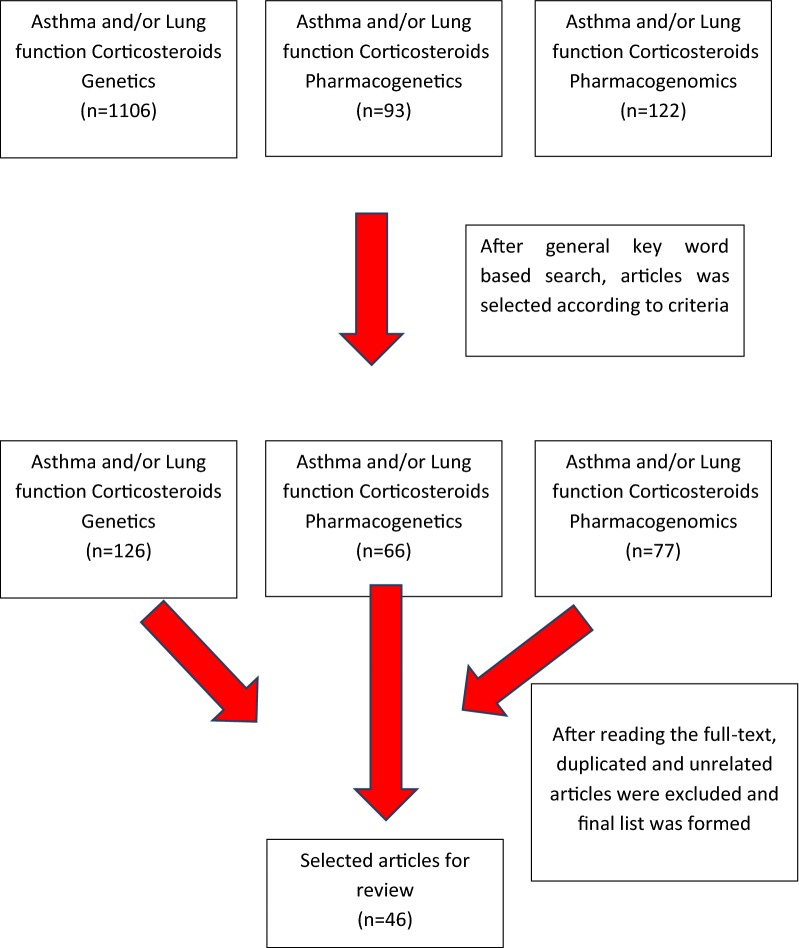
Flowchart for the inclusion of ICS pharmacogenomic studies

A search using the keywords “Asthma and/or Lung function, Corticosteroids, Genetics”yielded 1106 studies; “Asthma and/or Lung function, Corticosteroids, Pharmacogenetics” yielded 93 studies; “Asthma and/or Lung function Corticosteroids Pharmacogenomics” yielded 122 studies. Of these, 46 met the inclusion criteria with 38 being candidate gene studies and 7 being GWAS and 1 being exome sequencing study (Fig. 1). Nine articles remained after excluding the studies before 2015 June.

From all the publications selected, the following data were extracted: ICS type and duration of treatment, genes and SNPs, outcome, study design, ethnic backgrounds of patients, sample size, age range and duration of the study. From GWAS, information regarding the in vitro or in silico analysis after the association analysis was extracted. This systematic review is reported according to PRISMA guidance [11].

## Novel pharmacogenomics studies

From 2015 to 2018, nine additional studies were identified through PubMed. Of these studies, three were GWAS and six were candidate gene studies.

### GWAS (Genome-wide association studies)

GWAS have provided the opportunity to detect novel pharmacogenetic variants related to ICS response by scanning a high number of genetic variants across the entire genome. In the systematic review by Farzan et al. [10], the results of the four GWAS that had been conducted prior to 2015 have been described in detail. These GWAS had identified four new loci to be associated with ICS response. These loci harbored the *GLCCI1* (glucocorticoid-induced transcript 1 protein), *T*, *FBXL7* (F-box and leucine-rich repeat protein 7) and *ALLC* (Allantoicase) *genes* [12–15]. SNPs within *GLCCI1*, *T gene*, and *ALLC* were associated with changes in the lung function and rs10044254 SNP within *FBXL7* was associated with changes in the asthma symptom scores. From the identified SNPs within these genes, rs10044254 was the only SNP that reached the genome-widesignificance threshold. SNPs within the *GLCCI1*, *T gene*, and *FBXL7*, were associated with ICS response in pediatric asthma populations and were conducted by the same research group [12–14]. These studies included Caucasians from the Single-Nucleotide Polymorphism Health Association-Asthma Resource Project (SHARP). Although the three GWAS all studied the SHARP population, the methods of the GWAS differed quite a lot with regard to sample size, study design, outcome measurements, and genotyping platforms.

Between 2015 and 2018, three additional GWAS were published [16–18], of which one was conducted by the SHARP group. Changes in lung function were considered as the primary outcome in two of these GWAS and asthma exacerbations were considered the primary outcome in one study. None of the new GWAS could identify the previously found genes/SNPs in candidate gene or GWAS.

In the GWAS by Wang et al. [16], 120 mild-to-moderate adult asthmatics were included. In this study, patients received different doses of glucocorticoids (125, 250, 500, 1000 mcg) and the dosage of ICS was increased each week. The hypothesis underlying this approach was that integration of pharmacodynamic properties of corticosteroids into pharmacogenetic GWASs containing drug responses to different doses may increase the statistical power of significant association detection [19]. For the first time, a mechanistic model was applied to analyse the GWAS data with a series of dose-dependent pharmacological phenotypic data and detected associations of genome-wide significance between dose-dependent pulmonary function response to ICS, and five loci: rs6924808 on chromosome 6 (p = 5.315 × 10^−7^), rs10481450 on chromosome 8 (p = 2.614 × 10^−8^), rs1353649 onchromosome 11 (p = 3.924 × 10^−9^), rs12438740 on chromosome 15 (p = 4.499 × 10^−8^), and rs2230155 onchromosome 15 (p = 1.798 × 10^−7^). These loci are mapped to candidate genes related to cellular functions [20, 21]. Asthmatics who were homozygous for the mutant alleles had 30–300% higher % Forced expiratory volume in one second (FEV1) values at an intermediate dose of glucocorticoids compared to the homozygotes for the wild-type alleles and heterozygotes for all of the associated SNPs (with the exception of rs6924808). Some of the SNPs were sensitive to a small change in low drug doses and some showed variation after larger dose changes. Significant associations were also demonstrated in three additional independent replication populations. Two SNPs, chr6 rs6924808 and chr11 rs1353649, displayed an increased significance level (p = 6.661 × 10^−16^ and 5.670 × 10^−11^) in the pooled analysis of first GWAS results with these three replication trials. This GWAS underlined the importance of the optimal ICS dose for individualized asthma treatment based on a patient’s genetic makeup.

The first GWAS that studied asthma exacerbations as a primary outcome was performed by Dahlin et al. in 2015. In this study, 806 asthmatic children with Caucasian ethnicity were included from two population-based biobanks [17]. The results of the GWASs performed in these two populations were meta-analysed. However, none of the SNPs from the meta-analysis met the threshold for genome-wide significance. The most significant result was reported for 6 SNPs (rs2395672 and rs279728, rs4271056, rs6467778, rs2691529, and rs9303988) within three different genes *CMTR1* (Cap methyltransferase 1), *TRIM24* (Tripartite Motif Containing 24) and *MAGI2* (Membrane Associated Guanylate Kinase). Among these, the rs2395672 SNP within the *CMTR1* gene had the smallest p value from the meta-analysis [joint Odds Ratio (OR) = 1.07, 95% CI 1.03–1.11; p = 2.3 × 10^−6^].

The latest and so far largest GWAS of ICS response has been published by Mosteller et al. [18]. In this study, 2672 patients (12 years and older) were included from seven randomized, double-blind, placebo-controlled, multi-center clinical studies that were performed in a total of 26 countries. Changes in FEV1 at week 8 and 12 following fluticasone furoate (FF) or fluticasone propionate (FP) treatment were considered as the study outcome. The analysis was performed with more than 9.8 million genetic variants (minor allele frequency ≥ 1%), and none of the SNPs reached the genome-wide significance threshold.

An overview of published GWAS is provided in Table 1.

**Table 1:** GWAS analysis of response to ICS

Referernces	Genotyping platform and number of SNPs after pruning	Discovery phase population	Study design and duration	Number of asthmatic patients	Medication in the discovery phase	Replication population	Definition of response	SNPs chosen for replication (gene, chromosome position)	Study outcome
Tantisira et al. [12]	Human Hap 550v3 Bead Chip (Illumina) 534 290 SNPs	Caucasian children (CAMP)	Clinical trial, 16 months	118 child parent trios	Budesonide 200 μg twice daily	Caucasian patients (n = 935) Children: (CARE trial, n = 101, fluticasone propionate 100 μg twice daily) Adults: (Adult study, n = 385, 1000 μg daily (increased up to 2000 μg if necessary)) (LOCCS, n = 185, fluticasone 100 μg twice daily) (SOCS/SLIC, n = 264, triamcinolone 400 μg twice daily)	Changes in FEV1 from baseline	rs37972 (*GLCCI1*,7p21.3)	In two replication populations patients homozygous forthe wild-type allele (C) hadapproximately 12% improvements in FEV1% compared with the 4% increasein TT carriers after 4–8 weeks of treatment with ICS (combined p = 7×10^−4^)
Tantisira et al. [13]	Affymetrix (Santa Clara, CA) using a Human SNP array 6.0 444 088 SNPs	Caucasians children: (CAMP and CARE trials) (n = 239) Adults: (ACRN trial) (n = 179)	Clinical trial, 6–8 weeks	418	CAMP: budesonide 200 μg twice daily CARE: fluticasone propionate 100 μg twice daily ACRN: Triamcinolone 400 μg twice daily	Adults (n = 407) Flunisolide 1000 μg once daily (increased to 2000 μg if necessary)	Changes in FEV1% pred from baseline	rs3099266, rs1134481 and rs2305089(*T gene*, 6q27)	Patients homozygous for the wild-type allele of all three SNPs had a two to threefold increase in FEV1%pred compared to homozygotes for the mutant allele Combined p values of study populations for the rs1134481, rs2305089 and rs3099266 were 1.57 × 10^−5^, 2.3 × 10^−4^ and 1.1 × 10^−4^ respectively
Park et al. [14]	Human Hap 550v3 Bead Chip or Infinium HD Human610-Quad Bead Chip (Illumina, San Diego, CA) 440 862 SNPs	Caucasian children (CAMP)	Clinical trial, 8 weeks	124	Budesonide 200 μg twice daily	Caucasian Children: (CARE, n = 77, fluticasone propionate 1000 μg daily (increased up to 2000 μg if necessary)) Adults: (LOCCS, n = 110, fluticasone 100 μg twice daily) (ACRN, n = 110, Triamcinolone 400 μg twice daily)	Self-reported asthma symptoms based on diary cards. Scores ranged from 0 (absent) to 3 (severe)	rs1558726 (*RMST*,12q21), rs2388639 (*LOC728792*) and rs10044254 (*FBXL7*, 5p15.1)	The combined p values of rs2388639, rs10044254 and rs1558726 SNPs for the pediatric CAMP and CARE subjects were 8.56 × 10^−9^, 9.12x10^−8^ and 1.02x10^−5^ respectively. Homozygotes for the mutant allele for rs10044254 had significantly poorer responses to treatment compared to the patients homozygous or heterozygous for the wild-type allele (increase of 1.14 (as median score) in homozygotes for the mutant allele versus 0.28 in homozygotes for the reference allele)
Park et al. [15]	Illumina Human 660 W BeadChip (Illumina, San Diego, USA) 430 487 SNPs	Korean adults with moderate severe asthma	Clinical trial, 4 weeks	189	1000 µg of fluticasone propionate daily	Same population with the discovery phase	Changes in FEV1%	14 SNPs within *ALLC* (from GWAS) and 11 additional SNPs in *ALLC* (2q35)	rs17017879, rs7558370, rs11123610, rs6754459, rs17445240 and rs13418767 were significantly associated with change in FEV1% (p value < 1.0 × 10^−5^)
Wang et al. [16]	Affymetrix 6.0 arrays 909 622 SNPs After pruning 266 944 SNPs were involved in the analysis	120 Mild-to-moderate adult asthmatics	Clinical trial, each dose of ICS was used for 1 week	120	Inhaled multiple different doses of glucocorticoids-budesonide (125, 250, 500, 1000 mcg), in which, each dose was used for 1 week and the dose was doubled for the subsequent week	The IMPACT trial (n: 225, mild, persistent adult asthma, open-label budesonide or prednisone as guided by the symptom-based action plan. The run-in and treatment phases both ended with a 14-day period of intense combined therapy) Salmeterol off corticosteroids (SOCS) and salmeterol ± inhaled corticosteroids (SLIC) trials include 79 and 106 adult asthma,respectively, at the end of the 6-week run-in period on ICS, the milder patients were allocated to SOCS and the more moderate patients allocated to SLIC	Changes in FEV1%	rs6924808 on chromosome 6 rs10481450 on chromosome 8 rs1353649 on chromosome 11 rs12438740 on chromosome 15 rs2230155 on chromosome 15	The following loci produce associations of genome-wide significancewith physiological response to glucocorticoid therapy;rs6924808 on chromosome 6 with wild-type allele C andmutant T (p = 5.315 × 10^−7^), rs10481450 on chromosome 8 withwild-type allele A and mutant T (p = 2.614 × 10^− 8^), rs1353649 on chromosome 11 with wild-type allele G and mutant A (p = 3.924 × 10^−9^), rs12438740 on chromosome 15 with wild-type allele C and mutant T (p = 4.499 × 10^−8^), and rs2230155 onchromosome15 with wild-type allele C and mutant T (p = 1.798 × 10^− 7^)
Dahlin et al. [17]	Illumina’sOmni2.5 Exome BeadChip (Illumina, Inc., San Diego, CA) BioVU (731,390 SNPs) PMRP (662,256 SNPs) were firstmergedandprunedtoobtain 740,924 commonautosomalSNPs. In final dataset 237,726 common, independentSNPswereincluded	BioVU at VanderbiltUniversityMedical Center in Tennessee	Patients had initiated ICS treatment prior to the exacerbation event	806 Caucasian asthmatic BioVU N = 369 PMRP N = 437	ICS (beclomethasone, budesonide, ciclesonide, flunisolide, mometasone, ortriamcinolone)	PersonalizedMedicineResearch Project (PMRP) at theMarshfieldClinic in Wisconsin	Asthmaexacerbations	*CMTR1* rs2395672 rs4271056 rs279728 *TRIM24* rs6467778, *MAGI2* rs2691529, rs9303988	Six novel SNPs associated with differential risk of asthma exacerbations (p < 10^−5^). Rs2395672 in *CMTR1*, was associated with an increased risk of exacerbations in both populations (OR = 1.07, 95% CI 1.03–1.11; joint p = 2.3X10^−6^). Two SNPs (rs2395672 and rs279728) were associated with increased risk of exacerbations, four SNPs (rs4271056 (*CMTR1*), rs6467778 (*TRIM24*), rs2691529, and rs9303988 *MAGI2*) were associated with decreased risk
Mosteller et al. [18]	2184 haplotypes from the 1000 Genomes Project > 9.8 million common genetic variants	2672 asthma patients (≥ 12 years)from 7 randomized, double-blind, placebo-controlled, parallel group, multi-center clinical studies in 26 countries	“8–12 week” randomized double-blind placebo controlled parallel group multicenter clinical trial	2672 asthma patients (≥ 12 years)	Inhaled fluticasone furoate (FF)- or fluticasone propionate (FP) treatment		FEV1change at week 8 and 12 following FF- or FP treatment		No genetic variant met the prespecified threshold for statistical significance
Leusink et al. [67] (Exome array)	Infinium Human Exome chip (Illumina, San Diego, CA, USA), version 1.1, which contains 242 902 variants For common SNP analysis: MAF ≥ 1%, 36,519 SNPs For rare SNP analysis: MAF < 1%. 24,944 SNPs	CATO study 110 children with asthma that was not well controlled despite ICS	2-year randomized clinical multicenter trial Participants were followed up for 2 years, with the symptom-free days in the 2 weeks beforeeach visit, FEV1%, airway hyperresponsiveness (AHR) to methacholine (MchPD20) every threemonths. Treatment dosage was adjusted according to the algorithm of the study	110 children with asthma	L1-100-μg Fluticasone L2-200-μg Fluticasone L3-200-μg Fluticasone and 100-μg salmeterol L-4 500-μg Fluticasone and 100-μg salmeterol L5-1000-μg Fluticasone and 100-μg salmeterol		FEV1%, AHR (Mch PD20) and ICS response outcomes measured by the increase or decrease of FEV1% and AHR		Strongest association for rs72821893 in *KRT25* with FEV1% (p = 3.75x10^−^5), Mch PD20 (p = 0.00095) and MchPD20-based treatment outcome (p = 0.006) The 17q12-21 region was found associated with FEV1%pred and AHR, and ICS treatment response

Table adapted from Farzan et al. [10]

### Evidence of functional activity of the previously identified genes and SNPs in GWAS

#### GLCCI1

*GLCCI1*is a protein-coding gene located within the chromosome 7p21.3. The role of *GLCCI1* as one of the target genes of glucocorticoids was described by Chapman et al. more than two decades ago. They showed an increased expression of this gene in two different cell lines upon the administration of dexamethasone [22]. Glucocorticoids can induce apoptosis in several immune-inflammatory cells such as eosinophils and lymphocytes. Therefore, researchers concluded that reduced expression of *GLCCI1* because of rs37973 could result in reduced apoptosis of these cells. Consequently, reduced apoptosis of these cells could decrease ICS efficacy. To validate the functional role of the gene and its SNP in asthma control, an in vitro study was performed using lymphoblastoid B cells derived from children in the Childhood Asthma Management Program (CAMP) study [12]. This study showed that dexamethasone significantly increased *GLCCI1* expression. Specifically, in patients homozygous for the mutant allele (G), the expression was significantly lower compared to homozygotes for the wild-type allele (A) in the presence of dexamethasone. Increased expression of the *GLCCI1* gene was associated with a better response to ICS. Furthermore, Hu et al. [23] in their mRNA analysis showed an increased expression in *GLCCI1* after ICS. Taken together, GG carriers appear to be less sensitive to corticosteroids at the cellular level.

#### FBXL7

*FBXL7*, located in chromosome 5, is a member of Skp1-Cul1-F-box complex which is involved in ubiquitylation and degradation of proteins in the cells [24, 25]. In-vitro studies show that FBXL7 induces cell apoptosis and its cellular abundance is regulated by FBX18, another SCF protein [26]. Therefore, increased *FBXL7* expression can induce cell and tissue injury. In fact, studies on lung epithelial cells show that overexpression of *FBXL7* results in mitochondrial damage [27]. FBXL7 mediates this function through a decrease in the amount another protein (survivin) involved in cell survival and apoptosis. To date, more than 50 F-box proteins have been identified and these proteins appear to play major roles in inflammation and immunity by interacting with each other [28, 29]. A study in murine lung epithelial cells showed that F-box family members interact with proteins that can induce cytokine release in immune cells [26]. In the GWAS of asthma symptoms by Park et al. [14], the variant allele of the rs10044254 SNP was associated with poor symptom control. A further in vitro analysis using immortalized B cells obtained from the CAMP participants demonstrated that the variant allele was associated with a decrease in *FBXL7* expression in response to dexamethasone. Although not statistically significant, increased dexamethasone-induced expression of *FBXL7* was associated with poor asthma symptom scores. Despite these promising findings, no further studies have tried to elucidate the role of these proteins in asthma control.

#### T gene

*T* gene, located within the chromosome 6, is a member of the genes containing the T locus as a common protein motif [30]. The product of the T locus seems to be involved in the development of all vertebrate organisms. Furthermore, *T* gene expression has been previously shown in healthy adult lung tissue. In the GWAS study by Tantisira et al., researchers performed a pathway analysis in order to unravel the potential influence of the T gene on response to corticosteroids. To that end, using the program GeneMANIA (http://genemania.org/), they focused on the T gene–NR3C1 (Nuclear Receptor Subfamily 3 Group C Member 1) interactions [13]. The results showed that the *T gene* is co-expressed with the *NRIP1*, *FOXA2*, and *TTPA* genes. The same analysis showed that these genes directly interact or are predicted to interact with NR3C1.

Considering the important role of corticosteroids in lung development, it has been suggested that alterations in corticosteroid-responsive genes during development mighthave an influence on bothasthma susceptibilityand treatment response. Previous studies have shown that a decrease in T gene expression inhibits chondrogenesis, the process of cartilage development, mediated by BMP2 and FGFR3 [31]. These two proteins have been shown to be associated with corticosteroids resistance [32, 33]. As suggested by Tantisira et al. [13], these findings might provide a mechanistic basis for the T gene in response to ICS. However, despite these findings no further studies have tried to explore the functional role of this gene in ICS response.

#### ALLC

The study by Park et al. [15], was the first study ever to report an association between the *ALLC* gene and a disease or treatment response. By performing in silico analysis, Park et al. reported correlations between the *ALLC* function and three SNPs (rs13418767, rs6754459, and rs13409104) in high LD with the most significant SNP (rs11123610) from the GWAS. Furthermore, using the TFSEARCH program, they showed that rs13418767 is a binding site for Sp1, a member of the Skp1-Cul1-F-box complex. A recent study that investigated the association between occupational exposure to pesticides and genome-wide DNA methylation sites found differential DNA methylation in the *ALLC* gene [34]. In patients with airway obstruction who were exposed to high doses of pesticides, two different CpG sites of the *ALLC* gene were significantly hypo-methylated. ALLC is an enzyme that has lost its uricolytic activity during vertebrae evolution. However, animal studies suggest rather than a non-functionality, there seems to be low expression level and low substrate affinity of this gene in animals [35]. There is limited knowledge regarding the role of this gene and its variations on asthma and treatment response. While *ALLC* located within chromosome 2 seems to have a lost function in humans, this chromosome harbors several genes that have been found to be associated with FEV1 [36, 37] and IgE levels [38].

#### CMTR1

*CMTR1*, located within the chromosome 6p21, is involved in mRNA capping [39]. Capping of mRNA stimulates the stability of mRNA and increases efficient mRNA translation [40]. Furthermore, the product of this gene (hMTr1) is involved in defense mechanisms against viral infections. Increased *CMTR1* expression has been found to be associated with T cell mediated immune response mechanisms in human peripheral blood mononuclear cells [41]. Using independent microarrays, Dahlin et al. [17], evaluated the expression level of the top genes identified in their GWAS. They collected nasal lavage samples from children during and 1–2 weeks after asthma exacerbations. The expression level of *CMTR1*, but not *TRIM24* or *MAGI2*,was significantly reduced 1–2 weeks after exacerbations. Since viral infections are the main causes of asthma exacerbations in children and expression of CMTR1 has been shown to be upregulated in children during asthma exacerbations [42], this gene and its product might be promising new therapeutic targets that treat viral infections.

### Candidate gene studies

In total 29 candidate gene studies were included in the systematic review by Farzan et al. [10]. In summary, SNPs within the *CRHR1* (Corticotropin Releasing Hormone Receptor 1), *GLCCI1*, *FCER2* [Fc fragment of IgE, low-affinity II, receptor for (CD23)], *NR3C1*, *STIP1*, and *TBX21*(T-box 21) were studied the most. However, despite the large number of studied SNPs (> 500) within 120 genes, the most consistent results were reported only for one SNP, rs28364072, within the *FCER2* gene. rs28364072 was significantly associated with all three outcomes (lung function, symptoms, and exacerbations) in pediatric asthma populations.

Between 2015 and 2018, six additional candidate gene studies were published. These studies attempted to replicate the previously studied markers within the *GLCCI1* [23, 43–45]; *ADRB2* (Adrenoceptor Beta 2) [46, 47], *NR3C1* [43], *CRHR1* [43], *17q21 locus* [48] and *TBX21* [43]. SNPs located in the *GLCCI1*, *NR3C1*,*and 17q21* were positively replicated in independent populations. An overview of replicated candidate gene studies is provided in Table 2 [23, 43–61].

**Table 2:** Replicated genes of ICS pharmacogenomic studies

References	SNPs	Study population	Design	MAF	Medication	Defination of response	Study outcome
Tantisira et al. [49]	131 SNPs genotyped in 14 candidate genes in the steroid pathway ALOX15 (4 SNPs) CRH (4 SNPs) CRHBP (5 SNPs) CRHR1 (17 SNPs) FCER2 (15 SNPs) GATA3 (9 SNPs) HSD11B1 (10 SNPs) IL18BP (7 SNPs) MAPK8 (4 SNPs) NFATC4 (11 SNPs) NR3C1 (16 SNPs) POMC (6 SNPs) POMC (6STAT3 (10 SNPs) POMC (6STAT5A (10 SNPs)	Caucasian children and adults: Adult study (Adults, n = 415) CAMP (Children, n = 201) ACRN (Adults, n = 224)	Three independent 6–8 week clinical trials	rs242941 0.3 (T) rs1876828 (T)	Flunisolide 1000–2000 μg once daily Budesonide 200 μg twice daily Tramcinolone acetonide 400 μg twice daily	Changes in FEV1% from baseline	(*CRHR1*, NM_004382), was associated with treatment response in all three populations Individuals homozygous for the variants manifested a doubling to quadrupling of the lung function response to ICS compared with lack of the variants (p values ranging from 0.006 to 0.025 for three asthmatic populations). rs1876828, rs242939, and rs242941, were each associated with ICS treatment response in both the Adult Study and CAMP. rs1876828, was also strongly associated with improved FEV1 over the 6-week triamcinolone treatment in ACRN adult asthmatics
Dijkstra et al. [50]	*CRHR1*(17q21.31) rs1876828 rs242939 rs242941	281 adult patients with symptomatic asthma under 45 years of age	Asthma cohort followed for 22 years, clinical trial (Netherland)	rs242941 0.4 (T) rs1876828 0.2 (T)	749 μg/days (426–1152) Mean daily dose of ICS was calculated to an equivalent daily dose of beclomethasone.	Immediate effect: Changes in FEV1 from baseline within 3–6 months Long-term effect:Rate of decline in FEV1 annually during 13.0 (7–19) years	*CRHR1* polymorphisms are not associated with immediate or long-term improvement in FEV1 by ICSs or with prevention of accelerated FEV1 decrease in adult asthma
Rogers et al. [51]	*CRHR1*-rs242941 *FCER2*-T2206C	311 children (5–12 years) African-American and Caucasian Children (CAMP)	CAMP 4-year clinical trial	*CRHR1* rs242941 0.26 (T) *FCER2* T2206C 0.26 (G) in Caucasians, 0.44 (G) in African-Americans	Budesonide 200 μg twice daily	Exacerbations: Emergency Department visits Hospitalizations Oral prednisone burst Lung function measurements: poor responders:change in FEV1 %pred: ≤ 7.5%	Lower bronchodilator response to albuterol and the minor alleles of RS242941 in *CRHR1* (OR 1.6, CI 95% 1–2.7, p = 0.05) and T2206C in *FCER2* (OR: 2.1, 95%CI 1.2–3.5, p = 0.006) are associated with poor lung function response The minor allele of rs28364072 in *FCER*2 was associated with recurrent exacerbations in white subjects (OR: 1.9 for minor allele, p < 0.05) but it did not reach significance in multivariate analysis
Mougey et al. [52]	*CRHR1* (17q21.31) rs1876828 rs242941	Caucasian children, adolescence and adults continuing ICS (n = 65)	16 weeks Clinical trial	rs242941 0.34 (T) rs1876828 0.15 (T)	Fluticasone 100 μg twice daily	Change in FEV1% pred Asthma symptoms: Slopes of plots of ACQ scores versus time	Minor allele of rs1876828 was associated with improvements in FEV1% pred (p = 1.89 × 10^−4^) Minor allele of rs242941 was associated with decrement in FEV1% pred (p = 2.07 × 10^−3^)
Keskin et al. [43]	*NR3C1*(rs41423247) *CRHR1*(rs242939, rs242941, rs1876828) *TBX21* (rs2240017) *GLCCl1* (rs37973, rs3099266, rs2305089)	82 children with asthma exacerbation	Single high dose ICS study in children with moderate-severe asthma exacerbation, Clinical trial	*NR3C1* rs41423247 0.8 (G) *CRHR1* rs242939 0.08 (G) rs242941 0.27 (T) rs1876828 0.87 (G) *TBX21* rs2240017 0.04 (G) *GLCCl1* rs37973 0.47 (G) rs3099266 0.39 (A) rs2305089 0.35 (A)	Single-dose Inhaled 4000 mcg Fluticasone propionate +Nebulized albuterol solution	Changes in FEV1 at 4th hour	Homozygosity for the G allele at rs41423247 of the *NR3C1* gene is associated with a higher improvement in FEV1 at 4 h in children with moderate-to-severe asthma exacerbation treated with high-dose ICS (p = 0.006) No genotype-related significant difference was observed in SNPs *CRHR1* gene rs242939, s242941,and rs1876828, TBX21 rs2240017; *GLCCl1* rs37973; and *T gene* rs3099266 and rs2305089 in FEV1 change
Hosking et al. [53]	*GLCCI1* (*7p21.3*) Rs37973 (A/G)	Non-Hispanic white adolescents and adults (n = 1916)	Pooled data of seven studies, six studies of 8 week trials and one 12-week clinical trial	0.44 (G)	Various doses of Fluticasone furoate ranged between 25 and 800 μg daily, Fluticasone propionate 100–500 μg twice daily	Changes in FEV1 from baseline after 8 weeks in 6 studies and at week 12 in one study	There was no significant association between changes in FEV1 and rs37973 genotypes
Izuhara et al. [54]	*GLCCI1* (7p21.3) Rs37973 (A/G)	Adult Japanese (n = 224)	Asthma cohort receiving ICS fot at least 4 years	0.44 (G)	ICS maintenance dose varied between patients	Annual decline in FEV1 30 ml/year or more	rs37973 GG was associated with a decline in FEV1 of 30 ml/year or more (estimated effect: 1.10: 0.02 to 2.18, p = 0.047) There was no association between rs37973 genotypes and the outcome, when decline in FEV1 was analyzed as a continuous variable
Vijverberget al. [55]	*GLCCI1* (7p21.3) Rs37972 (T/C)	North European children and young adults BREATHE (n = 1037) PACMAN (n = 431) PAGES (n = 323)	Meta-analysis of three pediatric asthma cohorts	BREATH:0.45 (T) PACMAN: 0.44 (T) PAGES: 0.41(T)	ICS maintenance dose varied between patients	Exacerbations: Hospital visits OCS use Poor asthma symptoms: ACT scores ≤ 19 ACQ-scores ≥ 1.5	There was no significant association between increased risk of OCS use, increased risk of asthma exacerbations and rs37972 genotypes
Hu et al. [23]	*GLCCI1* (7p21.3) Rs37972 (T/C) Rs11976862, (A/G) Rs37973 (A/G)	Chinese population of 182 asthmatic patients and 180 healthy controls The association of GLCCI1 variations with ICS response was analyzed in 30 mild-to-moderate asthmatics	Case control: study 24 SNPs of GLCCI1 were genotyped in 182 asthmatic patients and 180 healthy controls. –Treatment trial: 2-week run-in period and maintanance ICS therapy for 12 weeks.	Rs37972 (T/C) Asthmatics:0.67 (T) Control: 0.12 (T) Rs11976862 Asthmatics: 0.07 (G) Control: 0.37 (G) Rs37973 Asthmatics: 0.55 (G), Control: 0.37 (G)	Inhaled fluticasone propionate (125 mg, twice a day)	Changes in FEV1	FEV1 change was significantly correlated with rs37972, rs37973 and rs11976862 at 4 weeks ICS (p = 0.021 for rs37972 and rs37973; p = 0.043 for rs11976862), at 8 weeks (p = 0.021, p = 0.025 and p = 0.035, respectively) and at 12 weeks (p = 0.040 for rs37972 and rs37973, p = 0.020 for rs11976862)
Xu et al. [44]	*GLCCI1* (7p21.3) Rs37973 (A/G)	Chinese population of 418 asthmatics	Inhaled fluticasone propionate/salmeterol combination (250/50 mg, twice daily) for the next 24 weeks. Follow-up visits occurred at 4, 12, and 24 weeks, and asthma control tests were reviewed and lung function tests were performed	0.49 (G)	Long term ICS treatment	Changes in FEV1	rs37973 was independently associated with poorer clinical therapeutic response to ICS Homozygotes for the wild-type allele who had a percent FEV1 change greater than 5% were more common than were homozygotes of the rare allele (rs37973, AA 67.01% vs. GG 49.49%, p < .05)
Rijavec et al. [45]	*GLCCI1* (7p21.3) Rs37973 (A/G)	208 Slovenian adults with atopic and nonatopic, mild-to-moderate persistent asthma	ICS (alone or in combination with a LABA, depending on the degree of asthma control Follow-up visits with spirometry testing after 3 months (short-term) and after at least 3 years (long-term) of ICS treatment	0.29 (G)	ICS or ICS + LABA, depending on the degree of asthma control	FEV1% change after ICS treatment (3 months) and at least 3 years Treatment was defined as successful when FEV1 decreased by < 30 mL/year	After 3 months of ICS treatment, the change in FEV1% was higher in patients with the GG genotype than in patients with the AG + AA genotype (7.5% vs. 4%, p = .049)
Szczepankiewicz et al. [56]	*GR* polymorphisms rs6190 rs41423247 rs6195 rs10052957	113 asthmatic children (6 to 18 years of age) (54 of children were with severe, difficult-to-treat asthma) 123 healthy control	Analysis of a relationship between the *GR* polymorphisms and poor response to glucocorticoids in asthmatic patients (Polish)	rs6190 0.03 (G) rs41423247 0.36 (G) rs10052957 0.39 (T) rs6195 0.08 (G)	ICS	The dose of ICS needed to achieve asthma control Worse response was defined as a necessity of taking high doses of ICS i.e. > 800 mcg of budesonide and > 500 mcg of fluticasone propionate	No association of *GR* polymorphisms with the dose of ICS needed to achieve asthma control was reported
Vijverberget al. [57]	50 tag SNPs were selected for 17 genes for screening: Ten genes were selected based on their involvement in the glucocorticoid (GC) receptor complex (*NR3C1*, *HSPCA*, *HSPA4*, *FKBP4*, *ST13*), GC transport (*SERPINA6*) or GC-mediated signalling (*CREBBP*, *TBP*, *NCOA3*, *SMAD3*) Seven genes were selected based on a previously reported association with asthma susceptibility, severity or asthma medication response (*ARG1*, *17q21* locus, *IL2RB IL18R1*, *PDE4D*, *HLA*-*DQ*, *BCL2*)	Children and young adults PACMAN (n = 357) BREATHE (n = 820) PAGES (n = 391) Validation cohorts; CAMP (n = 172) GALA II (n = 745) PASS (n = 391)	Meta-analysis of three cohorts	>0.2	Based on BTS guidlinesfortreatment step 2: SABA + ICS step 3: SABA + ICS + LABA step 4: SABA + ICS + LABA + LRA	Exacerbations: hospital visits Oral corticosteroid (OCS) use in the previous year	In a meta–analysis of six studies: *ST13* rs138335 remained associated with an increased risk of asthma-related hospital visits and OCS use in the previous year,; OR = 1.22 (p = 0.013) and OR = 1.22 (p = 0.0017) but did not passed Bonferronicorrection test None of the other genes including *NR3C1*, were associated at a nominal level with an increased risk of exacerbations in asthmatics using ICS in the three cohorts
Tantisira et al. [49]	*TBX21* rs2240017 (H33Q)	701 children aged 5–12 years with mild to moderate asthma enrolled in CAMP	CAMP, 4 year clinical trial	Minor allele frequency of H33Q: 4.5% no minor homozygotes were detected	Budesonide 200 μg twice daily	Bronchial Hyperreactivity (Methacholine) (PC20)	3.5-fold greater mean increase in log-transformed PC20 for methacholine after four years of inhaled budesonide in asthmatic children with glutamine variants when compared with either H33H homozygotes or in dividuals not taking ICS (p = 0.0002)
Ye et al. [46]	*ADRB2* (G16R A > G) *ADCY9* (I772 M T > C) *NK2R* (G231E G > A) *TBX21* (H33Q C > G)	53 mild-to-moderate adult asthmatics	Asthmatics adults (Korean) 5–12 weeks ICS treatment, Clinical trial	MAF of four selected polymorphisms were > 5	5–12 weeks of ICS	Asthma control status and FEV1	*NK2R* G231E G > A and *TBX21* H33Q C > G polymorphisms were significantly associated with asthma control status at 5–12 weeks of ICS treatment (p = 0.041, power = 81.419% and p = 0.006, power = 98.564%, respectively) The *NK2R* G231E G > A polymorphism also showed significant associations with the mean changes in FEV1% during 12 weeks of ICS treatment (p < 0.05)
Lopert et al. [59]	*TBX21* (17q21.32) rs9910408	208 adult patients with atopic and non-atopic, mild to moderate persistent asthma	3 years clinical trial	rs9910408 0.38 (G)	Treatment with ICS for 3 years (alone or in combination with long-acting beta agonists (LABA),according to achieved asthma control)	According to the response to ICS therapy patients were divided into ‘‘poor’’ and ‘‘good’’ responders BHR:Poor response was defined as an increase of PD20 for methacholine that was smaller than one doubling dose compared to the initial PD20 Lung function: Poor response decrease in FEV1 by more than 30 ml/year Asthma control: Poor response was defined as less than a three-point increase in the ACT score after at least 3 years of treatment Asthma-related quality of life: Poor response was defined as less than a 16-point increase from the initial AQLQ score	The frequency of AA genotype was significantly higher in good responders (p = 0.049) This genotype related response was even more evident in the subgroups of non-smokers (p = 0.008) and in non-atopic patients (p = 0.009) AA genotype was overrepresented among good responder saccording to changes in FEV1 in the subgroups of non-smokers (p = 0.013) and in non-atopic patients (p = 0.048)
Tantisira et al. [60]	*FCER2*(19p13.3) rs889182 rs2287867 rs12980031 rs8110128 rs4804773 rs7249320 rs2277989 rs1042428 T2206C rs4996974	311 children (CAMP) African-American and Caucasian children	CAMP 4-year Clinical trial	T2206C 0.26 (G) in Caucasians,-0.44 (G) in African-Americans	Budesonide 200 μg twice daily	Severe asthma exacerbation risk	Relative risk, expressed as hazard ratios, for exacerbations in those homozygous for the T2206C mutant allele were (3.95; 95% CI 1.64–9.51); and (3.08;95% CI 1.00–9.47).more likely to have a severe exacerbation compared to all other T2206C genotypes in both white and African-American children
Koster et al. [61]	*FCER2* (19p13.3) rs28364072 (A/G)	Caucasianchildren PACMAN (n = 386) BREATHE (n = 939) CAMP (n = 311)	PACMAN& BREATHE: Asthma cohorts CAMP: 4-year clinical trial	PACMAN 0.27 (G) BREATHE 0.26 (G)	For BREATHE and PACMAN: ICS maintenance dose varied between patients CAMP: Budesonide 200 µg twice daily	Exacerbations: Emergency department visits Hospitalization Asthma control: ACQ- scores Respiratory symptoms Asthma-related sleepdisturbances Asthma-related limitations in Daily activities Additional (airway) medication use during the preceding 12 month	The rs28364072 variant was associated with increased risk of asthma-related hospital visits in the meta-analysis (OR 2.38, 95% CI 1.47–3.85, p = 0.0004) The variant was associated with increased risk of uncontrolled asthma measured by ACQ scores (OR 2.64, 95% CI 1.00–6.98) and was associated with increased daily steroid dose (OR 2.46, 95% CI 1.38–4.39)
Berce et al. [68]	*ORMDL3* (17q21) rs2872507 (G/A)	-213 asthmatics who were regularly treated with ICSs	In asthmatics who were regularly treated with ICSs, spirometry was repeated after 4–6 weeks of treatment. Bronchial hyperreactivity was assessed with a methacholine challenge test	0.39 (A)	4–6 weeks of ICS: Children < 12 years of age: 200 mcg of fluticasone dry powder daily Children > 12 years of age: 400 mcg fluticasone dry powder daily	Changes in FEV1	Asthmatics with genotype AA had an 11.1 ± 16.0% mean increase in FEV1 after 4–6 weeks of ICS, compared with 4.6 ± 9.6% in GG homozygotes (p = 0.0463) Carriers of A allele had a 8.5 ± 13.8% mean increase in FEV1 after ICS treatment, which was significantly higher than 5.5 ± 10.7% in asthmatics with G allele (p = 0.0150)
Farzan et al. [10]	17q21 locus rs7216389 (C/T)	14 PiCA (Pharmacogenomics in Childhood Asthma) populations (4529 steroid treated children and young adults) Ten PiCA studies included patients with non‐Hispanic European origins, two included Hispanic patients, one African American, one East Asian patients	the association between variation in the 17q21 locus, and asthma exacerbations despite ICS use retrospective reporting of exacerbations in the observational cohort studies Prospective study in CAMP population	0.54–0.81 (T)	ICS	Asthma‐related hospitalizations/emergency department visit (ED) (ii) short courses of oral corticosteroid (OCS) use reported by the parent/child at the study visit or based on completed study questionnaires	In the meta‐analysis of 13 studies, rs7216389 was statistically significantly associated with asthma‐related ED visits/hospitalizations, (summary OR per increase in risk allele: 1.32, 95% CI 1.17–1.49, p < .0001, I2 = 3.9%) In the meta‐analysis of the nine studies, the rs7216389‐T was statistically significantly associated with an increased risk of OCS use/high‐dose ICS (summary OR per increase in variant allele: 1.19, 95% CI 1.04‐1.36, p = .01, I2 = 22.8%)

Table adapted from Farzan et al. [10]

### Replicated genes and SNPs

#### *GLCCI1* (glucocorticoid induced 1)

By 2015, three candidate gene studies had studied *GLCCI1* in order to replicate the findings of a GWAS published by Tantisira et al. [12]. In short, the *GLCCI1* rs37973 was positively replicated only in one of the three candidate gene studies. The positive replication study included 224 adult asthmatic patients of Japanese ethnicity. The SNP was significantly associated with an annual decline in FEV1 of 30 ml/year or greater [54]. After 2015, four additional studies, including one study by our own research group, have assessed the association between rs37973 SNP and ICS response [23, 43–45].

In a Chinese population of 418 adult asthmatics receiving 24 weeks of ICS treatment, rs37973 was independently associated with lower ICS response measured with FEV1 changes [44]. Percentages of homozygotes for the wild-type allele who had > 5% FEV1 change were more common than homozygotes of the rare allele (AA 67.01% vs. GG 49.49%, p < .05) [44].

In another Chinese study, Hu et al. evaluated the association of 24 SNPs within the *GLCCI1* gene with ICS response. They included 180 healthy individuals and 182 adult asthmatic patients from Chinese Han population. In line with the previous findings, patients homozygous for the G allele had significantly poorer improvements in their lung function compared to the heterozygotes and homozygotes for the A allele after 12 weeks of treatment with ICS [23]. Contrasting findings were shown in a study in 208 Slovenian adults with atopic and nonatopic asthma. FEV1% predicted was higher in patients with GG genotype than patients with the AG or AA genotype; after 3 months treatment FEV1% predicted change: 7.5% in patients homozygous for the G allele versus 4% (patients with AG/AA genotype), p value: 0.049. After 3 years of treatment, similar effects were observed; FEV1 %pred 7% (GG) versus 3.5% (AG/AA), p-value: 0.041) [45].

However, in a study we performed in 82 Turkish children with moderate-severe asthma exacerbation, we were unable to show any association between FEV1 increase after 4 h of single-high-dose ICS and variation at *GLCCI1* rs37973, rs3099266, rs2305089 genotypes [43].

Therefore, seven candidate gene studies have tried to replicate the results of the original GWAS. Interestingly, three studies that included East Asian populations (Japanese and Chinese) could positively replicate the results of the GWAS. Although the exact definition of the outcomes was different (annual decline in FEV1 and improvements in FEV1), all three showed that the G allele was associated with poor lung function outcomes. Interestingly, the minor allele frequency of rs37973 in East Asian patients was comparable to patients of Caucasian ethnicity. However, in contrast to the non-Hispanic white subjects in the GWAS, where LD was almost perfect between rs37972 and rs37973, in the study by Hu et al. this LD was far from perfect.

#### NR3C1

Glucocorticoids bind to their receptors in the cytosol and after the transfer of glucocorticoid receptor complex to the nucleus, they regulate expression of the genes involved in the inflammatory pathways [62, 63]. Therefore, due to its central role in glucocorticoid signaling pathway, *NR3C1*, the gene encoding GR, has been the center of attention in numerous pharmacogenomics studies [63, 64]. By 2015, two studies had investigated the role of SNPs in the *NR3C1* in glucocorticoid sensitivity and response. However, only one study in adults hadfound an association between anSNP within this gene (rs41423247) and response (defined by changes in FEV1 predicted) to prednisolone but not ICS. In-vitro and in vivo models have shown that the G allele of the rs41423247 locus at the *NR3C1* gene was associated with hypersensitivity to glucocorticoids [65, 66].

Recently, our group reported a significant association between rs41423247 and improvements in FEV1 after 4 h upon a single high-dose of ICS (4000 µg FP) in 82 Turkish children with moderate-to-severe asthma exacerbations [43]. Children with the GG genotype at rs41423247 had a higher improvement in FEV1 [24.2% (11.5–36.3)] compared to those with CG + CC, [7.9% (6.1–24.6) (p = 0.006)].

#### 17q21locus

17q21 is the most consistently identified locus associated with asthma onset in children and severe asthma, and it has also been linked to ICS response. By 2015, two studies examined the association between SNPs within the 17q21 locus and response to ICS [67, 68], though different variants were studied. A Slovenian study showed that asthmatic children treated with ICS showed a better improvement in FEV1% when they were homozygous for the AA genotype at rs2872507 at 17q21 compared to patients with AG or GG genotypes (13.3% vs. 7.0% vs. 4.9% respectively). In addition, a post hoc exome array analysis of Dutch children who participated in the Children Asthma Therapy Optimal (CATO) trial, showed that several variants in the 17q12-21 locus were found nominally associated with treatment response to ICS (based on FEV1% improvement or BHR during ICS treatment). The strongest association in this region was found for rs72821893 in *KRT25.*

In 2018, one of the largest pharmacogenomics studies [48] so far was performed by the international Pharmacogenomics in Childhood Asthma (PiCA) consortium [69]. In this study, more than 4000 children were included from 13 different studies. Genetic variation at position rs7216389 in the17q21 locus was found to be associated with an increased risk of OCS use and asthma-related hospitalizations/ER visits despite ICS use.

## Discussion

In the past 3 years, nine additional pharmacogenomics studies on ICS have been published, of which three GWAS. Although there is few overlap between identified variants and applied methodologies vary largely, in vitro and/or in silico analyses provide additional evidence that genes discovered in GWAS (e.g. *GLCCI1*, *FBXL7*, *T gene*, *ALLC*, *CMTR1*) might play a direct or indirect role in asthma/treatment response pathways.

Candidate gene analysis, on the other hand identified five SNPs within four genes (*CRHR1*: rs242941, rs1876828; *GLCCI1*: rs37973; *FCεR2:* rs28364072 and *TBX21:* rs2240017) that were positively replicated at least once. In line with the previous systematic review by Farzan et al., most consistent results were obtained for *FCεR2* (rs28364072) and *TBX21*(rs2240017) [46, 51, 58, 60, 61]. The only gene that showed a positive association in both GWAS and candidate gene analyses was *GLCCI1* [12, 23, 44, 45, 54]. However, it should be noted that this may be partly due to the fact the candidate gene studies were undertaken after identification of *GLCCI1* in a GWAS analysis.

Consistent significant associations were replicated between *FCεR2* rs28364072 and poor ICS response measured by exacerbations in two long follow up childhood studies [60, 61], asthma symptoms measured by ACQ in one long follow up childhood study [61], and lung function in one study [51] in asthmatic children. Moreover, supporting clinical findings, functional data related with *FCER2* provided a mechanistic basis for the observed associations with severe exacerbations. This variation in *FCER2*, T2206C, was associated with decreased *FCER2* expression and can adversely affect normal negative feedback in the control of IgE synthesis and action [60]. Moreover, it has been shown that the highest IgE levels were found in subjects both homozygous for the T2206C variant and taking ICSs, demonstrating a significant steroid-genotype interaction. This may actually be connected with the observation that higher IgE levels are associated with increased frequency of exacerbations [70], and hospitalizations [71, 72] in children with asthma.

Both studies investigating *TBX21* H33Q and ICS response showed significant association measured by two different outcomes: improvement in BHR in one childhood study of 4 years duration [58], and asthma control status in a 5–12 weeks adult study [46]. Since BHR and asthma control level are related to the quality of life in asthma patients and prognosis of asthma, genetic variation in *TBX21* may be important for asthma phenotypes. This finding was further supported by a functional study that showed that the *TBX21* variant increases T helper 1 and decreases T helper 2 cytokine expression compared to wild type [58]. However, we were not able show any association between TBX21 H33Q and FEV1 4 h after single-high-dose ICS in children with moderate-severe asthma exacerbation [43]. This difference may be due to different outcome parameter or different study design or different doses and duration of ICS.

Asthma is a very heterogeneous disease with various phenotypes and underlying disease pathways. Recent studies have emphasized the importance of defining the correct phenotype in successful asthma treatment. A good characterization of patients as well has a standardized definition of treatment response is therefore extremely important. Since most pharmacogenetic studies are underpowered, collaboration is inevitable. Novel consortia, such as the Pharmacogenomics in Childhood Asthma (PiCA) consortium, are emerging [69, 72] and are able to conduct large-scale GWAS meta-analyses, while performing sensitivity analyses for specific subgroups. This might lead to novel insights on the generalizability of findings between different populations, as well as more power to identify novel genetic variants.

In order to increase the utility of the pahramacogenomic studies in asthma the data that have emerged from GWAS, candidate gene, and mechanistic candidates should be evaluated together. This approach will allow the discovery not only of pharmacologic agents that directly target the disease asthma but also other pathways and biomarkers that are indirectly associated with or increase the risk of the disease such as atopy, eosinophils, bronchial hyperreactivity, pulmonary functions, and NO [73].

Pharmacogenomics may produce very important information for the practicing clinician. Predicting the drug response based on genetic testing has implications with respect to not only providing the best treatment but also preventing the adverse events that may be associated especially with higher doses and systemic CS [74, 75]. On a larger scale it may have tremendous effect on pharmacoeconomics by decreasing unnecessary medication use as well as by having a positive impact on the cost of the disease, as has been shown in other disease areas [76]. Even though ongoing international attempts such as “Ubiquitous Pharmacogenomics (U-PGx)” project “An Horizon2020 Program to Drive Pharmacogenomics into Clinical Practice” that is going on in seven European countries [77] holds promise, we are far from using pharmacogenomics data in clinical asthma practice. In contrast to long-acting beta-2 agonists [78], response to corticosteroids might be too complex to be mainly driven by a few genetic variants. Nevertheless, pharmacogenomics studies still might provide useful insights in underlying pathways or identify novel drug targets, especially when combined with other-omics layers (e.g. epigenomics, transcriptomics, microbiomics, breathomics) or assessing the interaction with the environmental factors using genome-wide interaction studies in well characterized patient populations [79]. Especially with the emergence of novel expensive biologics for patients with a poor response to ICS, it is of great importance to assess at an early stage which patients have an intrinsic poor response to ICS and will be eligible candidates for these novel targeted treatments. Genomics might, at least partly, help to answer this question.

## Abbreviations

ICS: inhaled corticosteroid
SNP: single nucleotide polymorphism
GWAS: genome wide association studies
SHARP: Single-Nucleotide Polymorphism Health Association-Asthma Resource Project
FEV1: forced expiratory volume in one second
FP: fluticasone propionate
FF: fluticasone furoate
CAMP: Childhood Asthma Management Program
GLCCI1: glucocorticoid-induced transcript 1 protein
FBXL7: F-box and leucine-rich repeat protein 7
ALLC: allantoicase gene
CMTR1: cap methyltransferase 1
TRIM24: tripartite motif containing 24
MAGI2: Membrane Associated Guanylate Kinase
NR3C1: nuclear receptor subfamily 3 group C member 1
CRHR1: corticotropin releasing hormone receptor 1
FCER2: Fc fragment of IgE, low-affinity II, receptor for (CD23)
ADRB2: Adrenoceptor beta 2
TBX21: T-box 21
